# Transgenic chickpea (*Cicer arietinum* L.) harbouring *AtDREB1a* are physiologically better adapted to water deficit

**DOI:** 10.1186/s12870-020-02815-4

**Published:** 2021-01-11

**Authors:** Alok Das, Partha Sarathi Basu, Manoj Kumar, Jamal Ansari, Alok Shukla, Shallu Thakur, Parul Singh, Subhojit Datta, Sushil Kumar Chaturvedi, M S Sheshshayee, Kailash Chandra Bansal, Narendra Pratap Singh

**Affiliations:** 1 Division of Plant Biotechnology, ICAR-Indian Institute of Pulses Research, Kanpur, 208 024 India; 2 Division of Basic Sciences, ICAR-Indian Institute of Pulses Research, Kanpur, 208 024 India; 3 Division of Crop Improvement, ICAR-Indian Institute of Pulses Research, Kanpur, 208 024 India; 4 Department of Crop Physiology, University of Agricultural Sciences, GKVK Campus, Bangalore, 560 065 India; 5 ICAR-National Institute of Plant Biotechnology, New Delhi, 110 012 India

**Keywords:** Genetic engineering, Transcription factor, *AtDREB1a*, Osmotic adjustment, Carbon isotope discrimination, Chlorophyll fluorescence, ETR, Yield, Phenotyping

## Abstract

**Background:**

Chickpea (*Cicer arietinum* L.) is the second most widely grown pulse and drought (limiting water) is one of the major constraints leading to about 40–50% yield losses annually. Dehydration responsive element binding proteins (DREBs) are important plant transcription factors that regulate the expression of many stress-inducible genes and play a critical role in improving the abiotic stress tolerance. Transgenic chickpea lines harbouring transcription factor, Dehydration Responsive Element-Binding protein 1A from *Arabidopsis thaliana* (*AtDREB1a* gene) driven by stress inducible promoter *rd29a* were developed, with the intent of enhancing drought tolerance in chickpea*.* Performance of the progenies of one transgenic event and control were assessed based on key physiological traits imparting drought tolerance such as plant water relation characteristics, chlorophyll retention, photosynthesis, membrane stability and water use efficiency under water stressed conditions.

**Results:**

Four transgenic chickpea lines harbouring stress inducible *AtDREB1a* were generated with transformation efficiency of 0.1%. The integration, transmission and regulated expression were confirmed by Polymerase Chain Reaction (PCR), Southern Blot hybridization and Reverse Transcriptase polymerase chain reaction (RT-PCR), respectively. Transgenic chickpea lines exhibited *higher* relative water content, *longer* chlorophyll retention capacity and *higher* osmotic adjustment under severe drought stress (stress level 4), as compared to control. The enhanced drought tolerance in transgenic chickpea lines were also manifested by undeterred photosynthesis involving enhanced quantum yield of PSII, electron transport rate at saturated irradiance levels and maintaining higher relative water content in leaves under relatively severe soil water deficit. Further, lower values of carbon isotope discrimination in some transgenic chickpea lines indicated higher water use efficiency. Transgenic chickpea lines exhibiting better OA resulted in higher seed yield, with progressive increase in water stress, as compared to control.

**Conclusions:**

Based on precise phenotyping, involving non-invasive chlorophyll fluorescence imaging, carbon isotope discrimination, osmotic adjustment, higher chlorophyll retention and membrane stability index, it can be concluded that *AtDREB1a* transgenic chickpea lines were better adapted to water deficit by modifying important physiological traits. The selected transgenic chickpea event would be a valuable resource that can be used in pre-breeding or directly in varietal development programs for enhanced drought tolerance under parched conditions.

**Supplementary Information:**

The online version contains supplementary material available at 10.1186/s12870-020-02815-4.

## Background

Chickpea is an important cool-season grain legume and a rich source of protein for vegetarians’ especially in developing countries and plays a vital role in ensuring nutritional security [1]. Chickpeas are predominantly grown under rainfed agro-ecosystem and conserved soil moisture is the primary source of available water for plant growth and development. Under rainfed conditions, if rainfall is inadequate, the crop often experiences increasing drought and high temperature stresses, as it progresses to the reproductive phase [2]. Terminal drought, thus, is one of the major constraints to chickpea production resulting in about 40–50% yield loss [3]. Therefore, there is an urgent need to enhance drought tolerance in chickpea for sustaining its productivity. Further, the global warming and predicted future climate change affecting precipitation, temperature, evapo-transpiration and other vagaries of weather will aggravate the severity of drought in years to come. Genetic mechanisms of drought tolerance in chickpea through various physiological and phenological adaptations have been well documented; however, the stability of drought tolerance across diverse agro-climatic conditions is very low due to high photo-thermoperiod sensitivity. Thus, adaptive mechanisms of chickpeas are ecosystem-specific. In order to improve drought tolerance and yield stability, genetic engineering approaches are essential which would invariably ensure expression of enduring traits in drought conditions. Drought tolerance is a complex trait, often governed by multiple genes involving many biochemical pathways. Recently, a “*QTL (Quantitative Trait Locus) hot-spot*” for drought tolerance has been identified on fourth pseudo molecule of assembled chickpea genome [4] and finely dissected to identify possible candidate gene(s) [5]. Further, resequencing of 429 lines of chickpea (cultivated and wild genotype) from 45 countries across the world reported 262 marker-trait associations (MTAs) and candidate genes for drought and heat tolerance [6].

Chickpea has narrow genetic base resulting from unique domestication pattern [6, 7] and hence, development of chickpea for enhanced drought tolerance is a very important endeavour. Genetic engineering offers the means to introduce specific traits related to drought tolerance and other traits in pulses [8, 9]. Genes known to be involved in stress response like transcription factors (TFs), protective proteins, osmolyte metabolism, reactive oxygen species (ROS)-scavenging proteins, signaling factors, post-translational modifications, small RNAs, epigenetic control of gene expression and hormonal networks are currently being envisaged for enhanced drought tolerance [10]. Dehydration responsive element binding proteins (DREBs) are important plant TFs that regulate the expression of many stress-inducible genes and play a critical role in improving the abiotic stress tolerance of plants [11, 12]. The TFs interact with dehydration responsive element (DRE)/C-repeat (CRT) *cis* element present in the promoter region of various abiotic stress-responsive genes and affect their regulation [13]. *AtDREB1a* are important APETALA2 (AP2)/ethylene responsive factor (ERF) group of TFs (isolated from thale cress, *Arabidopsis thaliana*) that induce set of abiotic stress tolerant genes involved in various abscisic acid (ABA) dependent as well as independent regulatory mechanisms [14, 15].

There are several reports in different crop species on enhanced abiotic stress tolerance utilizing *DREB* gene viz. freezing tolerance in *Arabidopsis* [16], water deficit stress in tomato [17], drought, low temperature and salinity tolerance in tobacco [18, 19], drought and salt stress tolerance in chrysanthemum [20], increased transpiration efficiency (TE) in peanut [21], drought tolerance in soybean [22, 23], and better root and shoot partitioning and higher TE in chickpea [24]. The diverse role of the *DREB* gene is aptly demonstrated in the reports pointing to its ability to influence multiple abiotic stresses by regulated or constitutive expression in transgenic plants. Morphological characterization of transgenic plants over-expressing *DREB* gene under constitutive promoter has been reported to affect growth pattern and hence use of stress-inducible promoters gained wider preference. Subsequently, regulated expression of *DREB* gene in rice [25], potato [26, 27], peanut [28, 29], cotton [30] and chickpea [24] have also been reported.

Here, we report the development, molecular characterization, detailed phenotyping of a transgenic chickpea event harboring *rd29A* driven *AtDREB1a* gene and compare grain yield under well-watered (WW) and water stressed (WS) conditions in Transgenic Containment Facility (PBSL1), to understand the role of *AtDREB1a* in adapting chickpea to water deficit conditions.

## Results

### Production of transgenic chickpea lines

In the present study, multiple shoots were induced from co-cultivated explants (cotyledons with half embryonic axis) of chickpea (*cv*. DCP 92–3) employing pre-standardized genetic transformation protocol. The kanamycin resistant shoots after 4–5 cycles of regeneration were grafted in pre-germinated chickpea rootstocks (*non-transformed*) in Transgenic Containment Facility (PBSL1) and could be established as mature fertile plants (Supplementary Figure 2). A total of 4031 explants were co-cultivated with *Agrobacterium tumefaciens* harbouring the *AtDREB1a* gene and total of four mature fertile primary transformants (T_0_) (E_5_, E_17_, E_19_, E_22_) were established with transformation frequency of 0.1%. Selfed seeds (T_1_) were harvested from all four established plants. In subsequent seasons, T_2_ seeds were harvested from PCR positive T_1_ plants for detailed molecular analyses. Further, seeds of one transgenic event (E_17_) were advanced to T_3_ stage for detailed phenotypic evaluation under dry-down conditions. Details of seeds harvested from all four events (T_1_ to T_3_ stages) are provided in Supplementary Table 1.

### Molecular analysis of transgenic chickpea lines

**Fig. 1:**
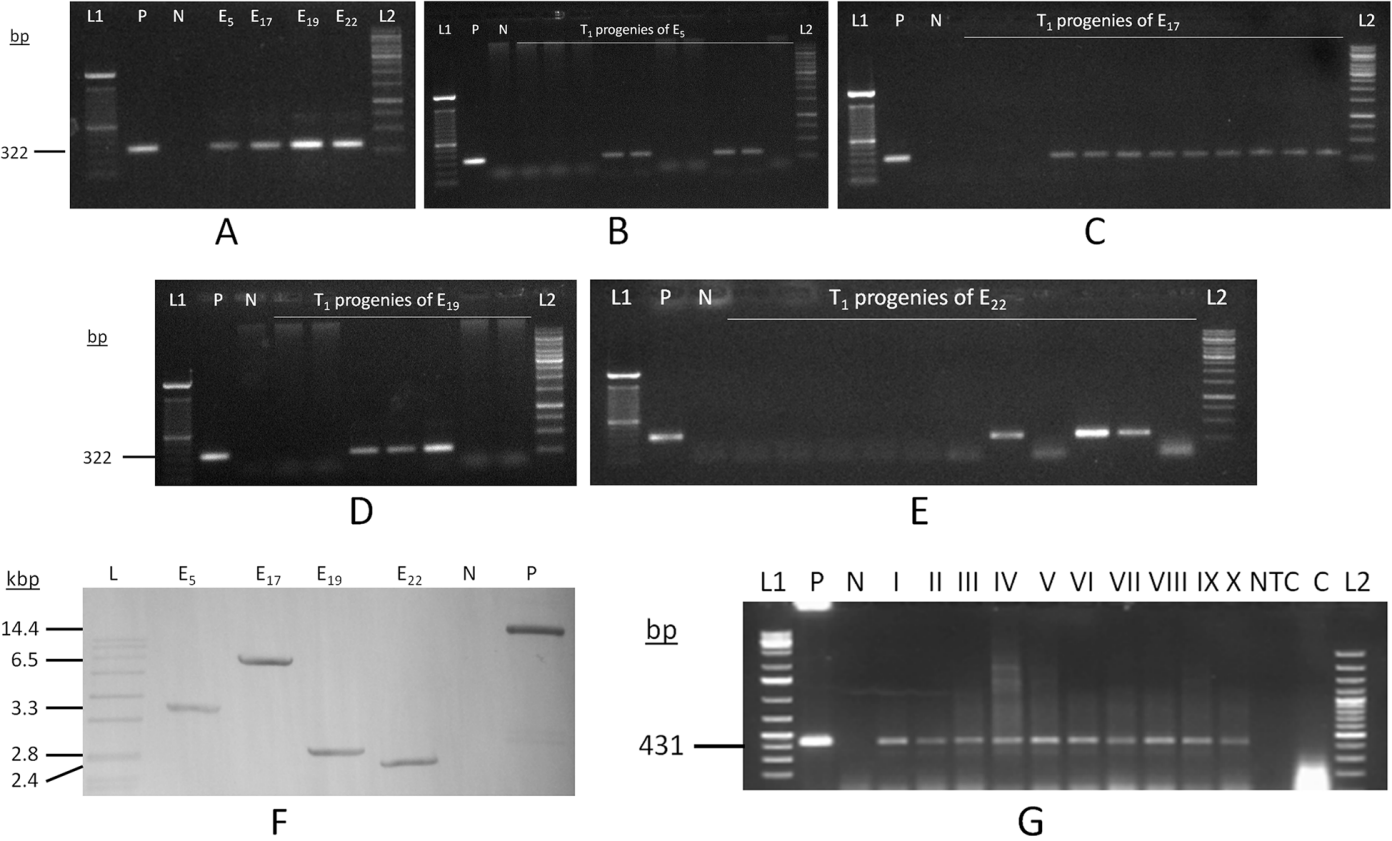
Molecular analysis of transgenic chickpea lines **a**: PCR analyses of four transgenic chickpea events (T_0_); **b**: PCR analyses of transgenic chickpea progenies (T_1_) derived from E_5_; **c**: PCR analyses of transgenic chickpea progenies (T_1_) derived from E_17_; **d**: PCR analyses of transgenic chickpea progenies (T_1_) derived from E_19_; **e**: PCR analyses of transgenic chickpea progenies (T_1_) derived from E_22;_ [L1–100 bp DNA ladder and L2–1 kb DNA ladder]; **f**: Southern blot analysis (L: DIG-labelled DNA ladder; I-IV: Four independent transgenic chickpea lines E_5_, E_17_, E_19_ and E_22_ (T1 stage); N: Non-transformed chickpea (DCP 92–3); P: Positive control (Binary plasmid). **g**: RT-PCR analysis (L1: 1Kb plus DNA ladder; P: Positive control; N: Negative control; I-IV: Transgenic chickpea lines (T_1_ stage); V–X: Transgenic chickpea lines (T_2_ stage); NTC: No Template Control; C: RNA as Template; L2: 100 bp DNA ladder) [Mean SM 11.8% and mean LWP −0.82 MPa]

PCR analysis with *AtDREB1a* gene and *nptII* gene specific primers indicated the presence of genes (650 bp and 322 bp respectively) in all the four established transgenic chickpea events (E_5_, E_17_, E_19_, E_22_) at T_0_ stage (Fig. 1a). PCR analyses of T_1_ progenies derived all the events indicated segregation pattern of the transgene (E_5_ (2:3), E_17_ (3:1), E_19_ (3:4) and E_22_ (3:8) (Fig. 1b, c, d and e) and *chi*-square test indicated 3:1 segregation pattern for E_17_ (Supplementary Table 2). Southern blotting performed from pooled chickpea progenies (T_1_ stage) of four events showed the presence and integration of *AtDREB1a* gene in unique position of chickpea genome (ca. 3.3 kb, 6.5 kb, 2.8 kb and 2.4 kb respectively) (Fig. 1f) (Supplementary Fig. 3). RT-PCR with gene-specific primers detected the presence of 431 bp amplification product in all the four events (T_1_ & T_2_) indicating the transcription of *AtDREB1a* gene in transgenic progenies, after stress (Fig. 1g) (Supplementary Fig. 4).

### Phenotyping of transgenic chickpea lines

**Fig. 2:**
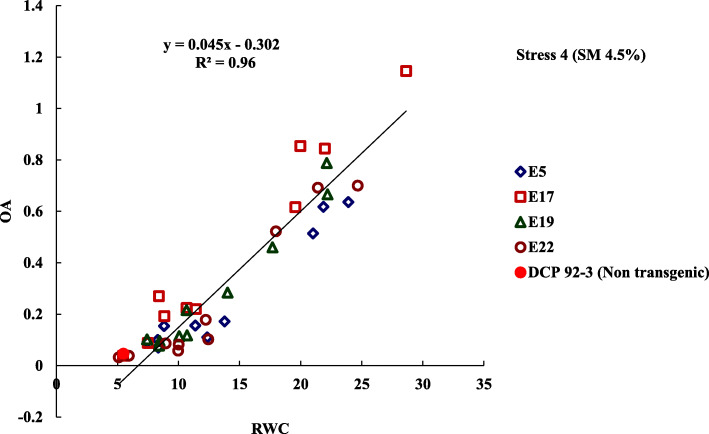
Association of OA with RWC observed in four transgenic events under Stress Level 4 (Soil moisture to 4.5%)

Preliminary phenotyping studies based on RWC and OA in the progenies of four transgenic events, invariably demonstrated improved expression of OA, when subjected to severe water stress (Stress Level 4) than their non-transformed counterpart, DCP 92–3 (control). Significant influence of drought responsive gene, *AtDREB1a* in modifying downstream biochemical pathways to impart tolerance under water limiting conditions, by improving plant water-relation including RWC were observed (Table 1) (Supplementary Table 3, 4, 5, 6). Out of four events tested, maximum level of OA expression were observed in progenies derived from Event E_17_, which also exhibited proportionately higher tendency to maintain RWC under severe water stress (Stress Level 4). Osmotic (OA) data of all derived lines of four transgenic events were regressed with corresponding RWC values to obtain linear relationship (R^2^, 0.95) between these two traits (Fig. 2).

**Table 1:** Mean Relative water content (RWC %) and mean osmotic adjustment (OA) of T_2_ progenies derived lines of four transgenic event at Stress Level 4

E_**5**_	Mean RWC (%)	Mean OA	E_**17**_	Mean RWC (%)	Mean OA	E_**19**_	Mean RWC (%)	Mean OA	E_**22**_	Mean RWC (%)	Mean OA
5.07	8.3	0.07	17.1	11.42	0.22	19.13	22.2	0.67	22.05	10.0	0.06
5.12	7.5	0.09	17.2	8.84	0.19	19.22	14.0	0.28	22.08	5.1	0.03
5.23	11.4	0.16	17.3	10.68	0.22	19.26	7.4	0.10	22.11	5.9	0.04
5.29	13.8	0.17	17.4	8.40	0.27	19.27	17.7	0.46	22.17	21.4	0.69
5.35	21.9	0.62	17.5	19.58	0.62	19.31	10.7	0.22	22.18	10.0	0.08
5.37	21.0	0.51	17.6	28.64	1.15	19.33	10.1	0.11	22.19	9.0	0.09
5.42	12.4	0.11	17.7	19.98	0.85	19.37	8.4	0.08	22.22	12.2	0.18
5.49	8.3	0.10	17.8	7.50	0.09	19.41	22.1	0.79	22.28	18.0	0.52
5.52	23.9	0.64	17.9	5.48	0.04	19.42	8.5	0.10	22.39	12.4	0.10
5.59	8.8	0.15	17.10	21.98	0.84	19.43	10.7	0.12	22.41	24.7	0.70
CON	5.5	0.05	CON	5.50	0.05	CON	5.5	0.05	CON	5.5	0.05
C.D.	4.6	0.22	C.D.	4.55	0.30	C.D.	4.66	0.23	C.D.	5.9	0.27
SE(m)	1.6	0.08	SE(m)	1.59	0.10	SE(m)	1.62	0.08	SE(m)	2.05	0.09
SE(d)	2.3	0.11	SE(d)	2.25	0.15	SE(d)	2.30	0.11	SE(d)	2.91	0.13
C.V.	25.96	66.1	C.V.	26.43	56.05	C.V.	27.562	60.926	C.V.	35.67	83.88
Range	5.5–23.9	0.05–0.64	Range	5.5–28.64	0.04–1.15	Range	5.5–22.2	0.05–0.79	Range	5.1–24.7	0.03–0.70

**Fig. 3:**
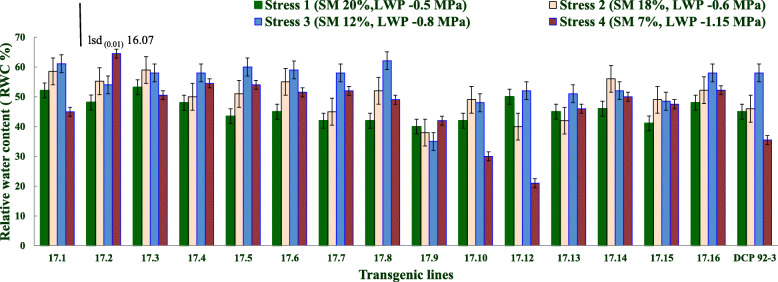
Leaf RWC of transgenic lines and control at four different stress levels (Stress level 1, 2, 3 & 4). Error bar represents the deviation from the mean values of RWC (lsd bar shows significance level at 1%)

All phenotyping studies were conducted using T_3_ progenies derived from sixteen T_2_ lines originating from one transgenic event (E_17_). Transgenic chickpea lines exhibited varying RWC status as the average soil moisture declined to 7% (stress level 4) from 20% (nearly at field capacity, saturated moisture, stress level 1). Transgenic chickpea lines exhibited both higher and lower leaf RWC at equal soil water stress level. With progressive development of drought to stress level 4 (soil moisture at 7%), progenies derived from two transgenic lines viz. 17.2 and 17.9, maintained higher RWC compared to their corresponding RWC values recorded at lower stress levels (stress 1 to 3). While other transgenic lines observed reduction in RWC at stress level 4, the transgenic lines 17.10 and 17.12 performed better. Transgenic lines maintained uniformly higher RWC with progressive increase in the water stress (1 to 4) (Fig. 3) (Supplementary Table 7).

**Fig. 4:**
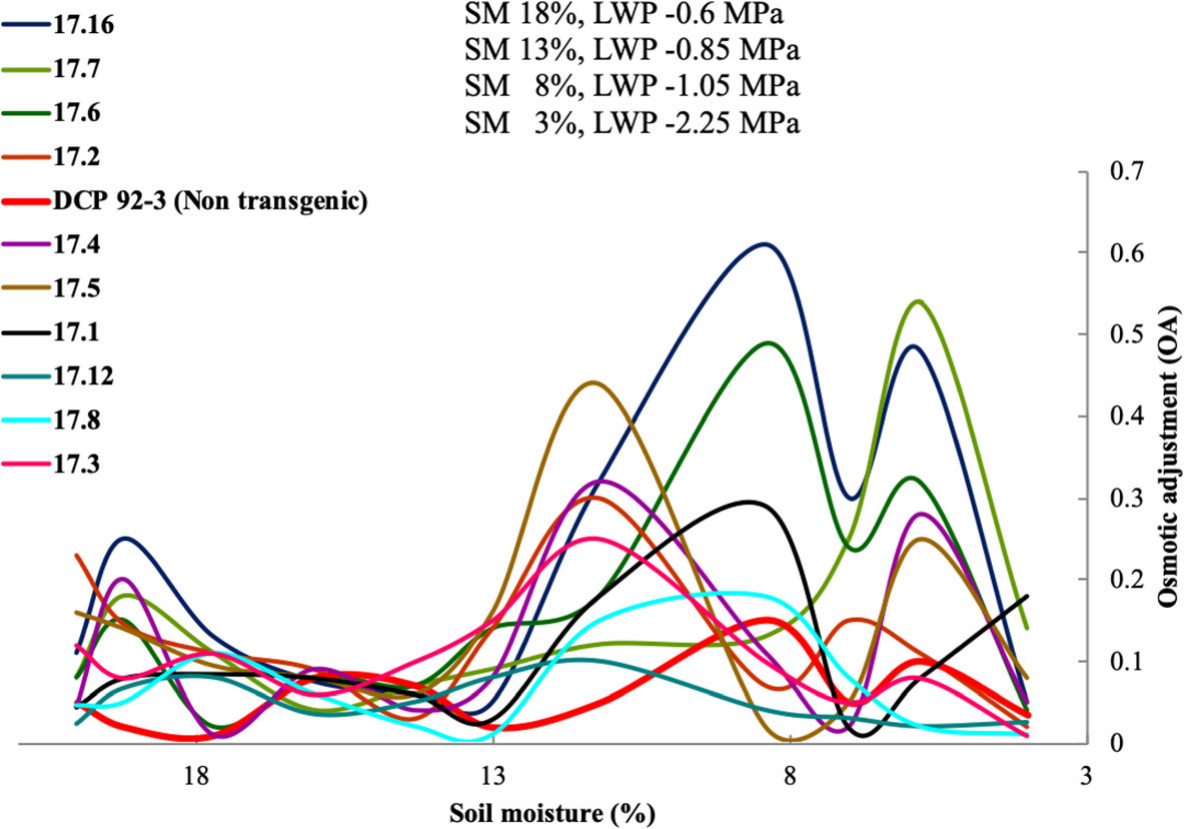
OA of transgenic chickpea lines with progressive decrease in soil moisture from field capacity to Stress Level 4

Transgenic chickpea lines exhibited much better OA as compared to control and significant OA was observed in the transgenic lines (17.2, 17.5, 17.6, 17.7, 17.15 and 17.16), only when the level of drought was severe with decline of soil moisture below 7% (stress level 4). However, there was periodic increase and decrease (polyphasic pattern) in OA during the entire stress period as SM declined from 22 to 4%. OA was prominently induced at relatively high stress, but did not increase consistently with decline in soil moisture. Notably, the non-transgenic DCP 92–3 (control) failed to show any significant OA under severe stress (soil moisture 7%), whereas transgenic lines (17.2, 17.6, 17.7 and 17.16) showed significant OA through enhanced accumulation of solutes (Fig. 4) (Supplementary Table 8).

Membrane stability indices decreased significantly in all the test transgenic chickpea lines including control when subjected to drought stress (− 1.03 MPa leaf water potential) for a longer period (stress 4), compared to non-transgenic control (− 0.53 MPa). Among the tested chickpea lines, progenies derived from 17.1, 17.2, 17.3, 17.6, 17.12, 17.16 exhibited higher MSI over control, under the same stress level (− 1.03 MPa), with maximum MSI observed in progenies derived from 17.6 at stress level 4. Overall, MSI ranged between 12.8 to 24% at stress level 4, while the values hovered between 24.1 to 49.3% at stress level 1 (Table 2) (Supplementary Table 9).

**Table 2:** Membrane stability index (MSI) of test transgenic chickpea lines and control under WS (Level 1) and WS (Level 4) with corresponding leaf water potential

Lines	Stress Level 1			Stress Level 4		
	Soil Moisture (%)	LWP (MPa)	MSI	Soil Moisture (%)	LWP (MPa)	MSI
17.1	18.5	−0.55	27.0	6.82	−1.42	22.2
17.2	19.2	−0.58	38.3	7.45	−1.25	21.8
17.3	18.0	−0.60	33.6	7.00	−1.11	20.8
17.4	19.5	−0.48	33.3	6.92	−1.05	13.5
17.5	20.0	−0.50	43.5	6.22	−1.22	14.6
17.6	20.1	−0.45	39.7	6.74	−1.00	24.0
17.7	19.8	−0.52	34.9	7.24	−1.15	16.2
17.8	18.8	−0.62	31.4	7.23	−0.98	17.0
17.9	19.6	−0.56	30.6	7.25	−1.15	16.6
17.10	20.0	−0.44	27.0	6.92	−1.21	15.8
17.12	20.4	−0.52	28.9	6.87	−1.11	17.6
17.13	19.6	−0.58	44.3	7.08	−1.08	12.8
17.14	20.5	−0.57	49.3	7.32	−1.11	13.6
17.15	20.4	−0.44	24.1	7.51	−1.18	13.7
17.16	20.2	−0.56	29.0	6.75	−1.20	17.0
Control	20.1	−0.48	27.0	6.88	−1.45	16.5
Mean	19.7	−0.53	33.9	7.01	−1.03	16.5
CD_0.05_	2.18	0.18	4.25	1.98	0.13	0.41

Values indicate mean of five replicates

**Fig. 5:**
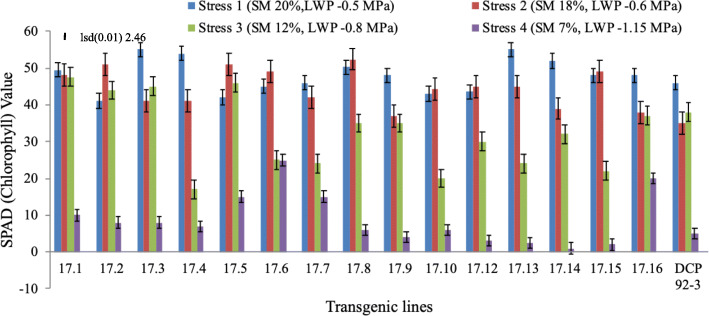
SPAD (chlorophyll content) values of leaves of transgenic chickpea lines and control at four different stress levels (Stress level 1, 2, 3 & 4). Error bar represents the deviation from the mean value of SPAD readings (lsd bar shows significance level at 1%)

The chlorophyll content, as estimated by SPAD meter, exhibited reduction in the chlorophyll invariably in all transgenic lines including control in response to drought, but the degree of reduction varied among the transgenic lines. In most of the cases, no reduction was noticed up to stress level 2 (LWP, − 0.6 MPa) but decreased significantly at stress level 3 onwards and drastically reduced at stress level 4 (LWP, − 1.15 MPa), in most of the transgenic lines. Notably, the two transgenic lines (17.6 and 17.16) have demonstrated better retention of chlorophyll even under severe stress (stress level 4) (Fig. 5) (Supplementary Table 10).

**Fig. 6:**
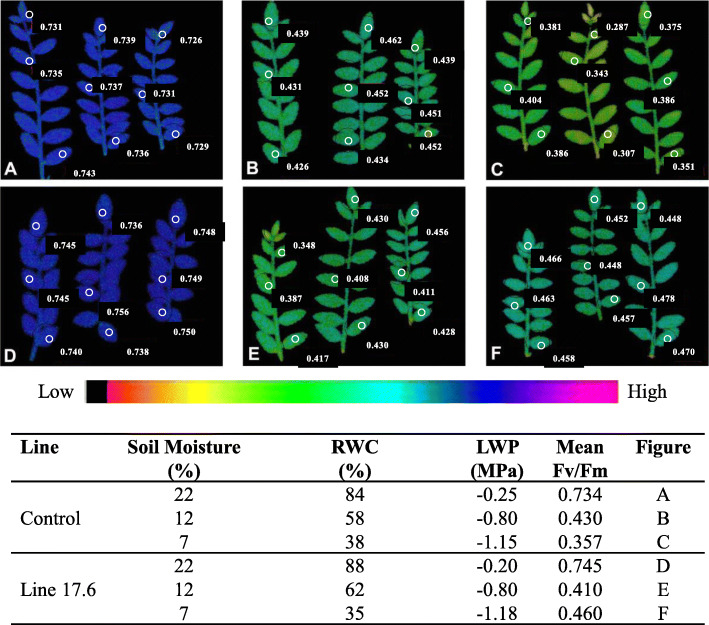
Images of photosynthetic quantum yield (Fv/Fm) of PSII under WW and WS in a transgenic chickpea line 17.6 and control. **a**, **b** & **c**- Quantum yield (Fv/Fm) images of control; **d**, **e** & **f**- Quantum yield (Fv/Fm) images of transgenic line 17.6. Corresponding values of SM, RWC and LWP were tabulated

The fluorescence images of leaves and corresponding values of quantum yield of PSII (Fv/Fm) of control and one transgenic line 17.6 were compared under WW and WS conditions. The chlorophyll fluorescence images of quantum yield of PSII indicate little or no differences between control and transgenic line, till RWC declined below 60% (LWP − 0.8 MPa) or even less. The mean values of quantum yield of PSII (Fv/Fm) of control were 0.734 (RWC 84%, LWP − 0.25 MPa), 0.44 (RWC 58%, LWP − 0.80 MPa) and 0.35 (RWC 38%, LWP − 1.15 MPa) (Fig. 6 a-c), whereas, mean values of quantum yield (Fv/Fm) of transgenic line 17.6 were 0.745 (RWC 88%, LWP − 0.2 MPa), 0.41 (RWC 62%, LWP − 0.8 MPa) and 0.46 (RWC 35%, LWP − 1.18 MPa), (Fig. 6 d-f) respectively. Three selections were made per leaf subjected to different level of WS. Overall, relative enhancement of photosynthesis in light adapted leaves of the transgenic line 17.6 was only observed during severe water stress subjected to LWP of − 1.18 MPa (RWC 35%). The color code of the images of quantum yield (Fv/Fm) represented the numerical values of quantum yield which differed significantly between control and the transgenic line 17.6 at RWC 35% (Fig. 6f). Thus, photosynthetic ability in terms of quantum yield (Fv/Fm) of PSII images could be precisely assessed to monitor the tolerance level of transgenic chickpea lines subjected to severe water stress.

**Fig. 7:**
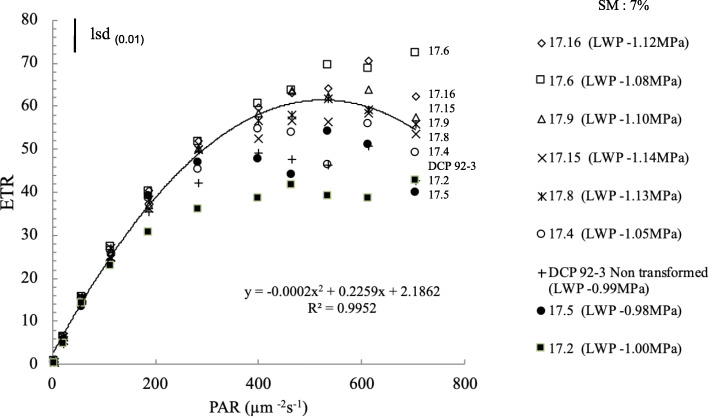
Photosynthetic ETR in response to increasing irradiance levels (PAR) in selected transgenic chickpea lines and control (lsd bar shows significance level at 1%)

The light-response of electron transport rate (ETR) of transgenic lines at stress level 4 when soil moisture declined to 7% (LWP − 1.0 MPa) indicated enhanced ETR at all irradiance levels, particularly > 400 μm m^− 2^ s^− 1^ (Fig. 7), as compared to control. Notably, transgenic lines (17.6, 17.8, 17.9, 17.14, 17.15 and 17.16) exhibited higher light saturation of ETR than control. Under light limiting conditions (200 μm m^− 2^ s^− 1^), ETR values of transgenic lines and control did not differ significantly, in spite of drought severity (− 1.0 MPa). However, most of the transgenic lines responded to high light saturation significantly with no photo-inhibition, at irradiance level of 700 μm m^− 2^ s^− 1^. This indicated that combined effect of drought and high irradiance modified the photosynthetic response in tested transgenic lines, as compared to control that exhibited photoinhibition at high irradiances (Fig. 7) (Supplementary Table 11).

**Fig. 8:**
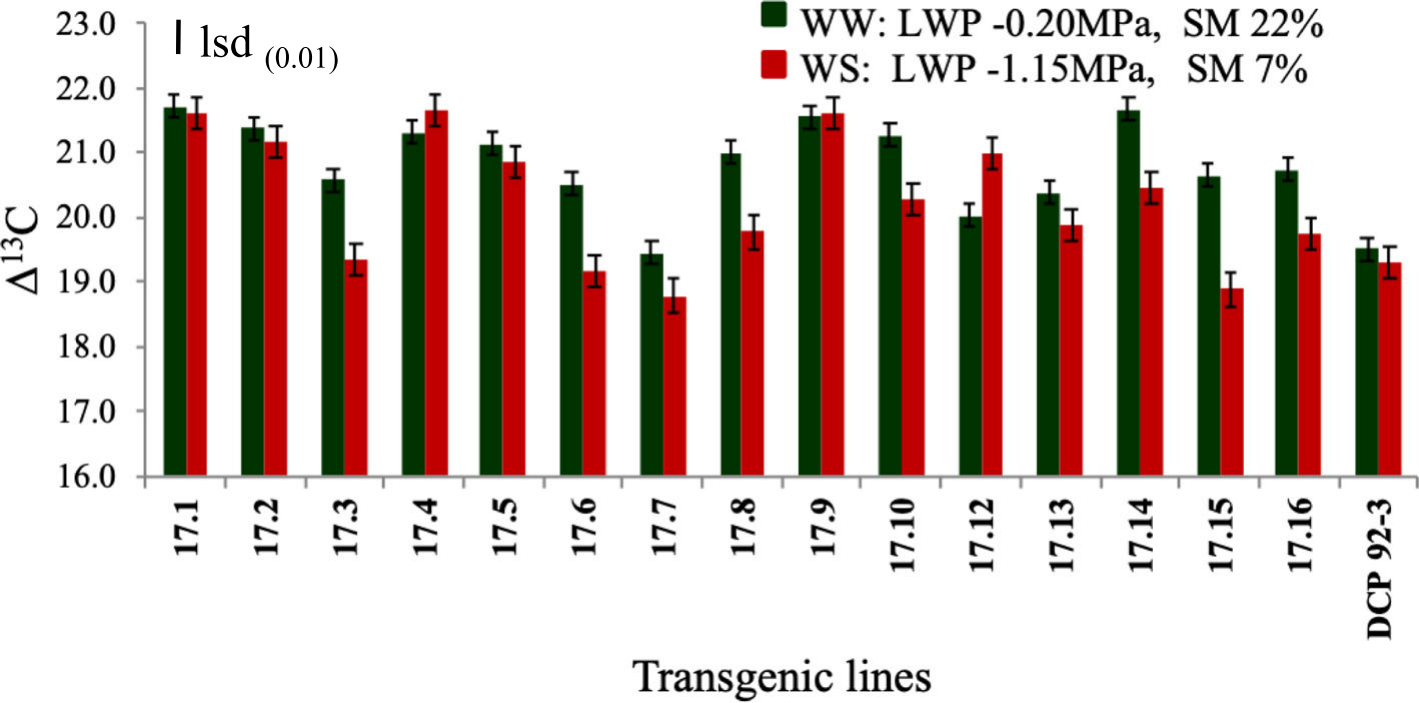
Histogram depicting CID values of transgenic chickpea lines and control under WW and WS conditions (lsd bar shows significance level at 1%)

With few exceptions, the CID in test transgenic chickpea lines and control exhibited decreasing trend, when subjected to water stress conditions (Stress Level 4, LWP − 1.15 MPa) as compared to well-watered conditions. However, significant reduction in CID values were obtained in transgenic lines (17.3, 17.6, 17.7, 17.8, 17.10, 17.15 and 17.16) compared to control indicating increase in WUE of the transgenic lines at stress level 4 (Fig. 8) (Supplementary Table 12).

### Yield of transgenic chickpea lines

**Fig. 9:**
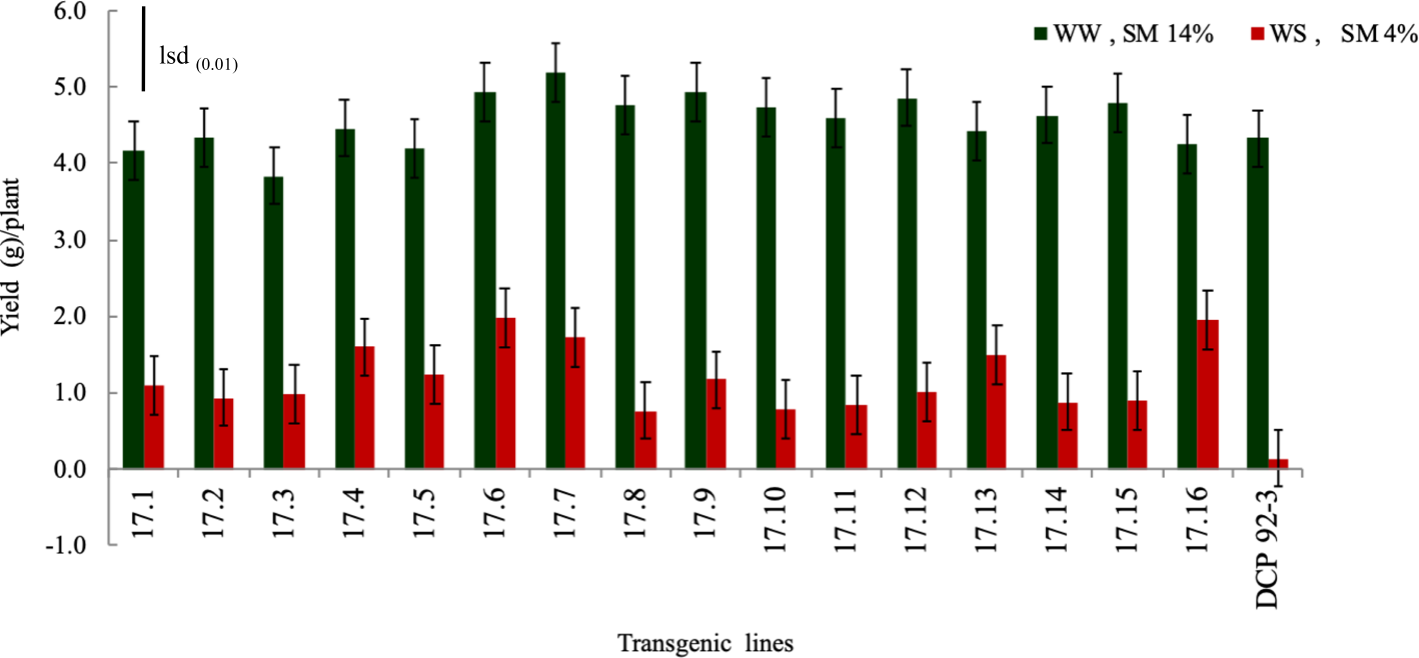
Histogram depicting the yield of the transgenic chickpea lines and control, under WW and WS conditions (lsd bar shows significance level at 1%)

No significant differences in seed yield were observed in the transgenic chickpea lines harboring *AtDREB1a* compared to their non-transgenic counterpart (control), under WW conditions. On the contrary, seed yield of the transgenic lines were significantly higher than control (*P* < 0.01), under water stressed condition (Fig. 9) (Supplementary Table 13), suggesting role of *AtDREB1a* in enhancing drought tolerance in the transgenic chickpea lines. Notably, transgenic chickpea lines 17.6 and 17.16 outperformed all test lines.

## Discussion

Tolerance of plants to abiotic stress relates to spatio-temporal expression of multiple genes affecting various physiological and biochemical pathways. Drought tolerance is considered to be polygenic character and manipulation of such trait requires interplay of many genes across various physiological pathways. TFs are master regulators that alter multiple-genes affecting several biochemical pathways, often referred as *regulons* [31]. Enhancement of drought tolerance by heterologous expression of TF, *AtDREB1a* has been widely reported in number of crops [17, 18, 21, 22, 24–26].

Chickpeas are important grain legumes, generally grown in sub-tropical regions of the world, often experience terminal water stress [32]. Drought tolerance in chickpeas could be improved through modification of several physiological mechanisms and *AtDREB1a* gene has been reported to enhance the drought tolerance in many crops [27–29, 33, 34]. The *desi* chickpea cultivar, DCP 92–3 is high input responsive, good agronomic background recommended for cultivation under irrigated conditions and is sensitive to drought. The cultivar, DCP 92–3 responds well in plant tissue culture and exhibit limited performance under drought conditions, and hence, the cultivar was used in the present study. Transformation efficiency obtained in the current study is 0.1%, however the efficiency can significantly be increased by modulating parameters like micro-injury to explants and LED-based light simulations, as reported [35]. Transgenic chickpea lines harbouring *AtDREB1a* gene driven by stress inducible promoter *rd29a* were developed and tested for various physiological parameters crucial to drought adaptation.

For phenotyping, plants were raised in pot size of 11.7 l capacity, fully saturated with water and allowed for full drainage until flow of water from pot bottom stopped and moisture content of pooled samples of soil from different depth were determined to be about 22%. During dry-down experiments, the soil moisture at middle zone was allowed to decline to about 7% by withholding irrigation, which is almost one third of field capacity. This was good enough to create desired level of drought in chickpea that resulted in minimal level of transpirable loss of water. The dry down experiments were designed as per the concepts developed earlier [36–38].

Chickpeas generally maintain high RWC with progressive drying of the soil. The RWC under water stress was regulated either through stomatal closure or plant’s inherent ability for OA through active accumulation of solutes. The control, non-transformed chickpea genotype, DCP 92–3 succumbs to stress; however, the transgenic chickpea lines exhibited enhanced OA to alleviate the drought stress. Initial RWC and OA based phenotyping pointed to the fact that osmolytes accumulation in leaf tissues, triggered by *AtDREB1A* expression and downstream activation lead to restoration of leaf water deficit under severe water stress (Stress Level 4). This in turn tends to maintain RWC in sustainable manner by mobilizing tissue reserve to growing sinks. Closure of stomatal openings upon progressive increase in water stress limits flow of water, as a result of diminishing transpiration pull, though apoplastic water might be available [39, 40]. If the leaf tissues are inherently capable by induced expression to accumulate osmolytes, it would have tremendous advantage to become water-saturated once the osmolytes concentration reaches a threshold level and to restore RWC. This induced cellular osmotic potential built-up might play a crucial role to confer enhanced tolerance and contribute to yield under water limited condition. Earlier studies in another pulse crop, pigeonpea indicated that OA maintained leaf longevity by preventing RWC dipping below the critical level of 32% [41]. Expression of *AtDREB1a* was associated with accumulation of osmolytes, in order to keep positive leaf turgor, maintenance of chlorophyll, increased RWC and enhanced MSI under stress, as reported earlier [42].

Correlating gene expression and RWC suggest that *AtDREB1a* has strong regulatory action on drought responsive genes in the developed transgenic chickpea lines. Transgenic lines 17.14, 17.15, 17.16, 17.2, 17.3, 17.4, 17.5, 17.6, 17.7 and 17.8 had significantly higher RWC compared to control at stress level 4 as the differences of RWC values in these lines are higher than 16.07 (lsd bar shown in Fig. 3). Notably, many lines have increased RWC under severe stress (level 4) compared to stress levels 2 and 3. The lines showing significantly higher RWC at stress level 4 had also increased OA in leaves. The higher OA tends to maintain higher RWC even under severe stress. The transgene, *AtDREB1a* may play significant role in activating the gene/s responsible for osmolyte accumulation in few transgenic lines leading to increased OA which was otherwise lacking in control plant, allowing faster decline in RWC in response to increasing intensity of the drought (level 4). It has been well documented that osmotic adjustment or increased solute accumulation maintain higher RWC or delays decline of leaf water potential with progressive increase in the drought stress [39, 40, 43].

Notably, chlorophyll content invariably decreased in transgenic chickpea lines, except for a transgenic line (17.6) exhibiting higher chlorophyll retention or greenness at stress level 4, as compared to other transgenic lines experiencing the same stress level. The decrease in chlorophyll content or forced maturity could be due to oxidative stress or pigment photo-oxidation resulting in degradation of chlorophyll, which is usually hastened when drought or other abiotic stress is imposed [44]. In order to ascertain the association of improved drought tolerance with photosynthetic activity, measurement of quantum yield of PSII and ETR were done. At 35% RWC, quantum yield of PSII was found better in transgenic line (17.6), possibly because of better chlorophyll retention and higher membrane stability. Similarly, better photosynthetic ability of transgenic lines was also reported in tomato and wheat [17, 45] suggesting conserved downstream signalling mechanism. Increased photosynthetic efficiency also concurs with earlier reports [46, 47]. The enhanced photosynthetic efficiency in stressed leaves of transgenic lines was also evident when high irradiance (≥700 μmol m^− 2^ s^− 1^) was imposed as additional stress. Interestingly, transgenic chickpea lines exhibiting high OA might be responsible for maintaining high photosynthesis under drought by virtue of their properties to act as osmoprotectants that help in maintaining membrane integrity during leaf dehydration. The observed ability of OA in transgenic lines under severe drought, possibly, imparts tolerance to stress, as well as maintain leaf turgor, membrane integrity and photosynthesis.

RWC has been reported to be the best indicator of plant water status in tomato, maize, wheat, etc. to differentiate tolerant and sensitive cultivars [48]. Distinguishably, the transgenic lines exhibited higher RWC from their non-transformed counterparts. The ability to maintain high RWC was evidenced by significant increase in the OA, as reported earlier [39]. On the contrary, cultivar DCP 92–3 exhibited reduced RWC to a greater extent, attributing to its inability to accumulate osmolytes or maintain desired level of OA.

Many physiological characters imparting tolerance to drought are not distinguishable or not expressed at milder drought conditions such as stress levels 2 and 3, therefore, desired consistency may not be very distinct at moderate stress. For example, in chickpea, osmotic adjustment was only exhibited in moderate to very severe stress (e.g. stress levels 3 and 4) and many of the associated processes such as chlorophyll retention (SPAD value) and photosynthesis was activated or sustained at severe stress. Hence, it seems appropriate to compare the parameters and differences in physiological parameters only when drought was intensified. In the study, severe drought stress (level 4) was found to affect photosynthesis by causing changes in chlorophyll or damages caused in photosynthetic apparatus (reduction in ETR and quantum yield) which is in agreement with earlier reports [49]. Similar results were reported in wheat, rice, barley and chickpea [50–53] under drought stress. In the present investigation, the higher amounts of osmolytes or increased OA observed in water stressed transgenic lines indicated an efficient mechanism of osmotic regulation, stabilization of sub-cellular structures (higher MSI), and cellular adaptation to drought which is in agreement with the earlier results [54, 55]. It is believed that drought induces metabolic changes and accumulation of low-molecular weight protective compounds, in addition, a large set of genes gets activated transcriptionally leading to accumulation of new proteins or amino acids, sugar alcohols or organic acids that contribute to osmotic adjustment which imparts tolerance to drought [56, 57].

The stability of the membrane under stress is one of the crucial factors for the plants to remain functional at cellular level. The loss of membrane integrity was evident when subjected to drought conditions. Transgenic chickpea line (17.6) exhibited least reduction in MSI, implicating conditioning for drought stress adaptation. The drought induced accumulation of compatible solutes as osmoprotectants is likely to be associated in imparting stability to the membranes as well as key enzymes and bio-molecules, which are prone to destabilization or subject to conformational changes under drought. Drought stress cause reduction in growth, leaf expansion and transpiration. Water stress induces ABA accumulation that result in stomatal closure to prevent loss of water through transpiration. At the same time, decline in Rubisco activity also results in limited photosynthesis [58, 59]. The stomatal closure leads to depletion of intracellular CO_2_ level causing generation of ROS components that interferes with normal metabolic process.

WUE of transgenic chickpea lines was also modified as measured by CID technique (^13^CO_2_/^12^CO_2_). Similar, modification of WUE of transgenic lines harbouring *DREB1a* TF targeting carbon fixing, *Rubisco* enzyme and TFs that regulate the photosynthetic and related metabolism under environmental stresses has been reported by other workers [60]. Higher TE or WUE in transgenic chickpea and peanut was also reported under drought stress [21, 24]. In another study significant correlation between TE or WUE and its surrogate traits in the groundnut transgenic lines (*rd29A*:*AtDREB1a*) under stress was established [61].

TFs are reported to influence photosynthesis and WUE through modifications of stomatal and non-stomatal components. The present study was confined to the measurement of photosynthetic efficiency through fluorescence imaging, exhibiting altered carbon metabolism/ETR indicating that transgenic lines enhanced photosynthetic response to drought through modifications of non-stomatal factors. In consistent with our findings, the transgenic peanut lines (*DREB1a*) exhibited increase tolerance to abiotic stresses through modification of WUE under WS condition [21]. Similar results were also reported in transgenic wheat lines [45].

Further, seed yield in transgenic chickpea lines were significantly related to osmotic adjustment, where, inter alia, transcription factor induced genes for osmolytes accumulation enhanced tolerance to drought (water deficit conditions). Our results concur with earlier reports of positive association of chickpea seed yield with osmotic adjustment under water stress in field experiments [62, 63]. As described earlier, the *desi* chickpea genotype DCP 92–3 was inherently lacking OA trait with lesser root biomass, and identified as *drought-sensitive* chickpea genotype. The osmotic adjustment is known to be poorly heritable character limiting improvement of drought tolerance and yield through conventional breeding. OA plays crucial role in maintaining cell turgor enabling plant to sustain photosynthesis under stress as evident from enhanced quantum yield of PSII and ETR. The photosynthetic product in case of chickpea under drought accumulated in the leaf as sucrose instead of starch due to drought-induced increase in the activation state of sucrose-phosphate synthase, as reported in earlier studies [39, 40]. As a result, the sucrose accumulates as osmolytes as well as facilitates transport of carbon source to growing seeds. Sucrose being the transportable form of sugar and its accelerated synthesis promote grain filling and the way osmotic adjustment plays double role: as maintaining turgor as well as facilitates transport of sugar. In addition, osmotic shock enhances the breakdown of reserve carbohydrates and nitrogen (proteins) and help remobilization of stored carbon and nitrogen to developing sinks and thus contributing to yield increase in chickpea. The present approach provides an opportunity to develop high-yielding enhanced drought tolerant chickpea cultivar by introgression of *AtDREB1a* in its parental line.

The chickpea crop experiences bi-phasic mode of water stress which includes transient mild drought during vegetative growth and terminal drought which is often severe during reproductive stage leading to substantial loss of grain yield, under water-limiting environment. The deep and profuse rooting system in chickpea largely has the ability to escape transient water stress experienced by plant, thus enabling to extract water efficiently from soil. The transient water stress is important for maintaining balanced plant water status, maintaining photosynthesis, transpiration efficiency and contributes towards increasing the biomass. The relation of modified root length and architecture towards higher water use in transgenic events has been clearly established in the earlier report [24]. However, as the crop approaches towards maturity, the density of functional roots in chickpea become limited due to high degree of lignifications of the roots with progressive decline in the soil moisture at terminal drought and as a result new root growth was restricted to sustain optimum plant water status. The present *AtDREB1a* transformed event essentially elaborated the previous work indicating the appropriate physiological adaptation mechanism to indicate how transcription factor modifies cellular constituents such as osmolytes accumulation which enable plant to conserve moisture within the system, maintaining cell stability through appropriate leaf water status and photosynthesis as phenotyped using chlorophyll fluorescence imaging indicating modifications of photosynthetic light reaction at chloroplast level. The present findings have added information on the advantage of osmolyte accumulation contributing efficient remobilization of photosynthates (osmolytes including sugars, amino acids, organic acids, nitrogenous products etc) in transportable forms to the growing sinks which are in high demand of carbon and nitrogen for development as it is evident by the higher seed yield in transformed events as compared to non-transformed plants. This conclusion was drawn on the basis of sharp decline in the osmolytes during a particular stage of high water stress after reaching a threshold level. These osmolytes products might have played a critical role in contributing yield improvement, adaptation to drought by maintain appropriate water status and supplement carbon and nitrogen towards functional roots under dehydrating soil. The transcription factor modifying the root architecture, length and transpiration efficiency as observed by earlier in transformed events could be beneficial where drought is not severe or, in other words in short duration crop that matures early before onset of severe terminal stress. Our findings differed with earlier report [24] which indicated expression of transcription factor targeting towards osmolytes increase, regulating the soil-plant water status and preventing disorganization of photosynthetic system at chloroplast level in alternate manner when the chickpea encounters severe terminal drought. Therefore, the current study has added information in the chickpea drought tolerance mechanism with sufficient novelty in terms of successfully establishing transgenic events with improved yield under drought.

## Conclusion

Improving drought tolerance in pulses and particularly in chickpea has been one of the major objectives towards stabilizing grain yield across different stress environments. The development of drought tolerant chickpea is also very important in the climate change perspectives. The present findings confirmed that stress-inducible expression of *AtDREB1a* gene in transgenic chickpea lines greatly enhanced drought tolerance, when severe drought stress was imposed. A number of adaptive physiological processes improved that conferred enhanced drought tolerance. Among various traits expressed in transgenic lines, the accumulation of osmolytes leading to osmotic adjustment in leaves seems to be very important as it improved water status, stabilized membrane integrity and maintained photosynthesis in coordinated inter-linked manner in response to drought. The selected transgenic lines would be a valuable resource that can be used in pre-breeding and direct variety development for improvement of chickpea drought tolerance under WS conditions.

## Methods

### Plant materials, plasmid construct and genetic transformation of chickpea

Breeders’ seeds of *desi* chickpea cultivar, DCP 92–3, were obtained from Seed Unit, ICAR-Indian Institute of Pulses Research, Kanpur. Seeds were sterilized with 70% ethanol for 5 min, followed by treatment with 1% sodium hypochlorite (v/v) for 3 min. The sterilized seeds were rinsed 3 times with sterile Milli-Q (Merck Millipore, Germany) water under aseptic conditions and soaked overnight at room temperature.

*Agrobacterium tumefaciens* strain, GV3101 [64] harbouring the binary vector pCAMBIA2300 (CAMBIA, Australia) containing the TF, *AtDREB1a* gene driven by stress inducible *rd29A* promoter was used for genetic transformation (Obtained from ICAR-National Institute on Plant Biotechnology, New Delhi) (Supplementary Fig. 1). This strain was grown overnight on a shaker at 28 °C in 50 ml of Yeast Extract Mannitol (YEM) broth containing 10 mg/l Rifampicin, 50 mg/l Kanamycin and 50 mg/l Gentamycin. The bacterial cells were harvested and the pellet was resuspended in 50 ml of half strength of Murashige-Skoog (MS) media pre-induced with 100 μM acetosyringone (Sigma Aldrich, USA).

*Agrobacterium tumefaciens*-mediated genetic transformation of chickpea was carried out as described earlier [65]. Cotyledons with half embryonic axis explants were prepared from overnight soaked seeds, by removing the seed coat and bisecting the embryonic axis longitudinally between cotyledons. The explants were dipped in prepared *Agrobacterium* suspension for 45 min and the infected explants were transferred on to co-cultivation media (MS salts, B_5_ vitamins, 1 mg/l NAA, 1 mg/l BAP, 0.8% Agar and pH 5.8). After 72 h of co-cultivation, the explants were sub-cultured to Shoot Induction Medium 1 (SIM1: MS Salts, B_5_ vitamins, 0.5 mg/l BAP, 0.5 mg/l Kinetin, 0.05 mg/l NAA, 10 mM MES, 100 mg/l Kanamycin, 250 mg/l Cefotaxime, 0.8% Agar and pH 5.8). After10–14 d, explants having green shoots were sub-cultured on to same media by removing cotyledons. After another 10–12 d, the explants surviving on selection were sub-cultured on to Shoot Elongation Medium, with reduced concentration of phytohormones (SEM: MS Salts, B_5_ vitamins, 0.1 mg/l BAP, 0.1 mg/l Kinetin, 10 mM MES, 200 mg/l Kanamycin, 250 mg/l Cefotaxime, 0.8% Agar and pH 5.8). The in vitro regenerated kanamycin resistant shoots were sub-cultured on to the same medium for 4–5 selection cycles of 10–14 d interval by separating the multiple shoots from each other. Green healthy shoots surviving kanamycin selection cycles were used as scions and grafted onto the pre-germinated rootstock, initially grown in soil matrix (soil: vermiculite: cocopeat) in Transgenic Containment Facility (PBSL1). The grafts were acclimatized and hardened to maturity and seeds (T_1_) were harvested from mature fertile plants (T_0_). Selfed seeds of transgenic chickpea lines were harvested and raised in PBSL1, for all subsequent generations (T_1_ through T_3_) reported in this study.

### Molecular analyses of transgenic chickpea lines

#### Polymerase chain reaction (PCR)

For PCR analysis, genomic DNA were extracted from 8 to 10 pinnules of leaves of 30–40 d old plants of four independent transgenic chickpea lines at T_0_ and subsequent stages, using the protocol described earlier [66]. The quantification of DNA samples were done using Biophotometer plus (Eppendorff, Germany) and each sample was diluted to 100 ng/μl. All the progenies of chickpea were screened for the presence of transgene using neomycin phosphotransferase II (*nptII)* (NPTF: 5’gattccgaagcccaacctttcatag3´ and NPTR: 5’tgccgaatatcatggtggaaaatgg3´) and *AtDREB1a* (AtDREB1AF: 5’atgaactcattttctgctttttctg3´ and AtDREB1AR: 5’ttaataactccataacgatacgtcg3´) specific primers. PCR was performed in 20 μl using 1X Taq Buffer, 250 μM dNTPs, 10 pM each primer, 200 ng DNA template and 1 unit of *Taq* DNA polymerase (Merck, India). Thermal cycling programme used was as follows: initial denaturation at 94 °C for 5 min followed by 35 cycles of 94 °C (denaturation) for 1 min, 60 °C (annealing) for 1 min and 72 °C (extension) for 1 min, final extension at 72 °C for 10 min and held at 4 °C in thermal cycler (G-Storm GS4 Tetrad Thermal Cycler, UK). PCR products were resolved on 1.0% agarose gel, stained with Ethidium bromide in 1X TAE buffer. Gel images were documented using gel documentation system (BioRad Gel Doc XR, USA). Chi-square test was also conducted to understand the segregation pattern of transgene in all four events at T_1_ stages, based on PCR analyses.

#### Southern hybridization

For Southern blotting, genomic DNA was isolated from leaves pooled from all PCR positive chickpea plants (T_1_ stage) derived from four independent transgenic chickpea events along with untransformed DCP 92–3 (control), using cetyl trimethyl ammonium bromide (CTAB) method [67]. The genomic DNA (15.0–20.0 μg) of transgenic lines was digested with *Bam*HI restriction enzyme (5 U/μg) that cuts the T-DNA once. Plasmid DNA (10.0 ng) and genomic DNA of untransformed DCP 92–3 (15.0–20.0 μg) were also processed simultaneously. The digested fragments of each line were electrophoresed in 1% agarose gel and blotted onto Hybond N+ membrane (Roche, USA). The digoxygenin (DIG) labelled probe specific to *rd29A* was synthesized using DIG labelling Kit with the chemiluminescent substrate, disodium 2-chloro-5-(4-methoxyspiro {1,2-dioxetane-3,2′-(5′-chloro) tricycle [3.3.1.1]decan}-4-yl)-1-phenyl phosphate (CDP-Star) (Roche Diagnostics GmbH, Mannheim, Germany). Labelling, hybridization, and detection were performed following manufacturer’s instruction (Roche Diagnostics GmbH, Mannheim, Germany). The signal was detected on an X-ray film after an exposure time of 10 min. The X-ray film was scanned using Pharos FX Plus Molecular Imager (BioRad, USA).

#### Reverse transcriptase-PCR (RT-PCR)

Total cellular RNA was extracted from the young leaves of all the transgenic chickpea lines (T_1_ and T_2_) (60 days after sowing) along with control (*cv*. DCP 92–3) before and after stress (withholding water for 10 d, corresponding to mean SM 11.8% and mean LWP − 0.82 MPa) using Spectrum™ Plant Total RNA Kit (Sigma-Aldrich, USA). Genomic DNA fragments were eliminated from total RNA preparation using on-column DNaseI digestion set, as per manufacturer instructions (Sigma-Aldrich, USA). The RNA was quantified using Nanodrop Biophotometer plus (Eppendorff, Germany) and an equal amount of RNA from each sample was used for the two-step RT-PCR reaction. The first strand cDNA synthesis was done using approximately 1 μg of total RNA (Verso cDNA Synthesis Kit, Thermo Fischer, USA) and the product obtained was further used for second-strand amplification. PCR was performed with house-keeping gene (actin) specific primer (ACTI1F: 5’acctcagcagagcgtgaaat3´ and ACTI1R: 5’ttgcaacaggacctctggac3´) and *AtDREB1a* gene specific primers (DREB1aF: 5’gtaagtgggtttgtgaggttagaga3´ and DREB1aR: 5’ttcatgattatgattccactgtacg3´) based on PCR amplification protocol described earlier. RT-PCR product was electrophoresed in 1.2% agarose gel and image was documented, as described earlier.

#### Phenotyping of transgenic chickpea lines

For initial phenotyping, T_2_ progenies derived from four independent transgenic chickpea lines (E5, E17, E19 and E22) were assessed in terms of RWC and OA in WW and WS conditions, as compared to control. A total of 130 seeds (30 seeds each derived from four transgenic events)] and 10 seeds of non-transformed chickpea (control) lines were set in complete randomized design (CRD) with five replications per treatment. The transgenic plants and control plants were divided into two groups. One group (65 nos: 60 (15 X 4) transgenic lines and 5 control lines) was maintained under WW condition and another group (65 nos) was subjected to WS by withholding water.

Seeds were sown in earthen pots (11.7 l capacity) filled with pre-weighed soil and compost mixture (10 kg), and measured quantity of water was given for germination. The pots were kept in natural-lit containment facility with maximum/minimum temperature 32 °C/20 °C. Irrigation was done when the soil moisture was reduced by 50% from initial level. Watering was done to maintain moisture level at field capacity until all the lines achieved desired heights with fully developed canopy. The stress level 1 reached after 45 days of withdrawal of irrigation from the stage when the soil was fully water saturated at field capacity (22%). Subsequently, the plants reached to level 2, 3 and 4 after 4th, 12th and 26th days, respectively from the stress level 1. The level of water stress was monitored through periodical measurements of soil moisture at 15 cm depth, using gravimetric method [68]. Soil samples were collected from 2 to 3 places using a hollow stainless steel cork borer (2 cm diameter and 20 cm long) by boring the potted soil deep down below 20 cm depth and slowly lifted out so as to retain only 10 cm below the upper soil surface. Pooled soil samples were weighed and oven dried at 110–120 °C for 3 days. Completely dried soil was re-weighed and percent soil moisture was calculated using the formula: [Fresh weight of soil – Dry weight of soil) /Dry weight of soil] X100). Individual plants were grown to maturity in the Containment Facility and samples for physiological parameters were collected at different stress levels and yield data at harvesting stage. Levels of stress have been classified as follows: Stress Level 1 (20% residual soil moisture); stress level 2 (18% residual soil moisture); stress level 3 (12% residual soil moisture); stress level 4 (7% residual soil moisture).

RWC was measured by taking the fresh weight (FW), turgid weight (TW) and dry weight (DW) of leaves of transgenic lines (T_2_ stage) as well as control plants at all four stress levels under WS condition and correspondingly in WW conditions. To determine the FW, fourth fully expanded leaf from the top of plants were collected and placed in a pre-weighed, hermetically sealed glass vial and weighed. Collected leaves were removed from the vial and the cut petiole was dipped in de-ionized water for 12 h at low light intensity to obtain the TW. The leaves were blot dried, then oven dried at 80 °C for 3 d to determine the DW. Finally, the RWC was calculated using the formula: RWC = (FW-DW)/(TW-DW) [69].

The OP was measured on the fifth leaf from the top of the test plants by detaching and freezing in liquid nitrogen for several days. The thawed samples were put in a 2 ml hypodermic syringe with a filter and the filtered cell sap was used for the measurement of OP using Wescor 5520 vapour pressure osmometer (Wescor Inc., Logan, UT, USA). OP at full turgor (OP_100_) was calculated using the following formula: OP_100_ = (OP x RWC)/100 [70]. Osmotic potential of dehydrating leaves were measured from 30th day onwards to physiological maturity at 150th day, and relationship of OP of leaves with stress days (progressive increase in water stress) was estimated. Osmotic adjustment (OA) was calculated from the difference in OP_100_ between leaves sampled at any given date and the least stressed OP under non-stressed irrigated condition, as described earlier [2]. Levels of OA in leaves of transgenic chickpea lines from field capacity to dry-down experiments (Stress level 1 through Stress level 4) were estimated. Further, detailed phenotyping studies were conducted with T_3_ progenies derived from 16 T_2_ lines (numbered 17.1 to 17.16) of one transgenic chickpea event (E_17_) [Total: 160 seeds (10 seeds each of 16 lines)] and 10 seeds of non-transformed chickpea (control) lines.

#### RWC, OP, LWP and MSI measurements

RWC and OP measurements were done as described in earlier sections. For LWP measurements, fully expanded fifth leaf from the top of the test chickpea plants were collected at all four stress levels. The leaves were excised and processed in pressure chamber using optimized protocol at the experimental site (PMS Instruments Company, USA) as essentially described [71], in terms of MPa.

The membrane stability index (MSI) was determined using electrolyte leakage (EL) method. The fourth leaf from the top of test plants were collected, washed using distilled water, surface dried and dipped in de-ionized water at 40 °C for 1 h. The electrical conductivity (EC) of tissue leachates was measured by using conductivity meter (Model: HI2300, Hanna, USA). The contents were incubated further by dipping the same leaf in deionized water at 100 °C for 1 h and EC was measured. The membrane stability index (MSI) was calculated by the formula: MSI =C1/C2, where C1= EC (EC μS) at 40 °C for 1 h and C2 = EC (EC μS) at 100 °C for 1 h [72].

#### Chlorophyll estimates

Chlorophyll status or “greenness index” (chlorophyll content in the leaves) was measured using SPAD meter (Soil Plant Analysis Development; Minolta, Model 502, Spectrum Technologies, Plainfield, Ill., USA). Fully expanded fourth leaves from the top of the plants of both groups (WW & WS) at all four stress levels were used for the study. SPAD meter measures the transmittance of the leaf in two wavelengths (600 to 700 nm and 400 to 500 nm) and SPAD values were recorded by pressing the light meter onto leaf surfaces. Higher greenness index/value relate to increased presence of chlorophyll in the leaves being monitored.

#### Fluorescence image analysis

Leaf samples of transgenic line 17.6 and control collected from both the groups (WW and WS) in fully turgid (LWP − 0.25 MPa and RWC 84–88%), moderate stress (LWP − 0.80 MPa and RWC 58–60%) and severe stress (LWP − 1.15 MPa and RWC 35–38%) were used for chlorophyll fluorescence studies [73]. With progressive imposition of drought, the photosynthetic response between the transgenic line and control were assessed rapidly using fluorescence Imaging System (Mess & Regeltechnik, Waltz, Germany). The dark-adapted leaves were exposed to weak modulated light with a frequency of 2 Hz /PAR (photosynthetically active radiation) 0.05 μmol with 100 μsec pulses followed by superimposition of saturation pulse of blue-enriched photon flux of 700 μmol for 100 m/sec to obtain *quantum yield* (Fv/Fm; Variable/Maximum fluorescence) and corresponding fluorescence images were captured. Subsequently, leaves were exposed to actinic light of 200 μmol photons m^− 2^ s^− 1^ for 2 min for light adaptation. Same saturated pulses were superimposed to obtain quantum yield in light adapted leaves. The selected area of leaves with corresponding quantum yield value obtained was compared with dark and different stress level for assessment of photosynthetic performance.

Light response of ETR representing the photosynthetic activity of leaves of all test transgenic lines and control under WW and WS were studied using inbuilt automatic software (ImagingWin, Walz-Imaging system, GmbH, Germany) employing irradiance range of 200–700 μmol m^− 2^ s^− 1^. The light curve and initial fluorescence values (F_o_ and F_m_ respectively) of the dark adapted leaves were used for calculation of ETR (ETR = Quantum yield x PAR × 0.5 x Absorptivity). Absorptivity describes the fraction of incident light which is absorbed and 0.5 indicates only half of the absorbed quanta is distributed to PS II (under steady state conditions). Light-curve of individual selection was obtained with increasing order of irradiance till ETR got light-saturated. Few samples were not in appropriate physiological state to include in ETR studies, hence experiment was restricted to few selected chickpea lines.

#### Carbon isotope discrimination (CID) measurements

For CID measurements, leaf samples from transgenic chickpea lines and control were collected at stress level 1 and 4. Harvested leaves were dried to 80 °C in a hot air oven for 3 d and finely powdered in a mill. Carbon isotope composition was determined on 1.0 mg sample with Isotopic Ratio Mass Spectrometer (IRMS, Model Thermo Finnigan, Bremen, Germany) at the University of Agricultural Sciences, Bangalore, India. The IRMS was interphased with a combustion device (Flash EA 1112) and worked on a continuous flow mode. Samples were applied to a flash combustion element analyzer (EA-1112, Carlo Erba, Milan, Italy). An adequate quantity of CO_2_ gas separated and purified by the element analyzer was introduced to an isotope ratio mass spectrometer (Delta XP Plus, Thermo Finnigan, Hamburg, Germany) to estimate the ratio of the isotopic composition expressed as δ13C (^13^CO_2_/^12^CO_2_) with the standard error of 0.1%. Standard potato starch calibrated against Pee Dee Belemnite carbonate was used for comparison. CID of the samples was calculated as described earlier [74].

#### Yield of transgenic chickpea line and control

Yield (g) of transgenic plants and control (*cv*. DCP 92–3) grown in well-watered (14% moisture at 15–20 cm depth) and water stressed (4% moisture at 15–20 cm depth) conditions were compared at full physiological maturity (150 days after sowing) in Transgenic Containment Facility.

### Statistical analyses

Experiment was set as Completely Randomized Design (CRD) with five replications each treatment and data was analysed using standard statistical software (Statistical Package for Social Sciences, SPSS, *ver*. 12.0). Statistical interpretations were derived based on analysis of variance (one way/two way ANOVA) and least significant differences (LSD) with significant level was tested using t-test at 1% or 5%.

## Supplementary Information

**Additional file 1: Sup Fig. 1.** T-DNA of the *AtDREB1a* construct. **Sup Fig. 2.** *Different stages of genetic transformation in chickpea (C. arietinum L.),* A. De-coated DCP 92–3 seed, B. Prepared CWHEA explant, C. Explants dipped in *Agrobacterium* suspension, D. Explants in Whatman filter paper, E. Co-cultivated explants in SIM 1 media, F. Germinated explants in SIM 2 media, G. Explant having multiple shoots, H. Kanamycin resistant shoot (Ready for grafting), I. Micrografting, J. Mature fertile plant. **Sup Fig. 3.** Full image of Southern Blot analyses (1F). **Sup Fig. 4.** Full image of RT-PCR of transgenic chickpea lines and control (1G). (PPT 3221 kb)

**Additional file 2: Sup Table 1.** Transgenic chickpea lines and Generation Advancement. **Sup Table 2.** Segregation of *AtDREB1a* in T_1_ progenies of four independent chickpea events. **Sup Table 3.** ANOVA for RWC and OA of transgenic event E5 (T_2_). **Sup Table 4.** ANOVA for RWC and OA of transgenic event E17 (T_2_). **Sup Table 5.** ANOVA for RWC and OA of transgenic event E19 (T_2_). **Sup Table 6.** ANOVA for RWC and OA of transgenic event E22 (T_2_). **Sup Table 7.** ANOVA for RWC of transgenic chickpea lines (T_3_). **Sup Table 8.** ANOVA for OP of transgenic chickpea lines (T_3_). **Sup Table 9.** ANOVA for MSI of transgenic chickpea lines (T_3_). **Sup Table 10.** ANOVA for chlorophyll content (SPAD values) of transgenic chickpea lines (T_3_). **Sup Table 11.** ANOVA for ETR of transgenic chickpea lines (T_3_). **Sup Table 12.** ANOVA for CID of transgenic chickpea lines (T_3_). **Sup Table 13.** ANOVA for seed yield of transgenic chickpea lines, under WW and WS conditions (T_3_).

## Data Availability

All data generated or analysed during this study are included in this published article. Transgenic chickpea materials reported in the study are available in Seed Repository, ICAR-Indian Institute of Pulses Research, Kanpur, INDIA (https://iipr.icar.gov.in/).

## Abbreviations

*AtDREB1a*: *Arabidopsis thaliana* Dehydration Responsive Element-Binding protein 1a
PBSL1: Plant Biosafety Level 1
RWC: Relative Water Content
ETR: Electron Transport Rate
OA: Osmotic Adjustment
OP: Osmotic Potential
MS: Membrane Stability
QTL: Quantitative Trait Locus
CID: Carbon Isotope Discrimination
TF: Transcription Factor
TE: Transpiration Efficiency
ROS: Reactive Oxygen Species
WW: Well Watered
WS: Water Stressed
SM: Soil Moisture
YEM: Yeast Extract Mannitol
MS: Murashige-Skoog
*nptII*: neomycin phosphotransferase II
PCR: Polymerase Chain Reaction
RT: Reverse Transcriptase
FW: Fresh Weight
TW: Turgid Weight
DW: Dry weight
LWP: Leaf Water Potential
WUE: Water Use Efficiency
PAR: Photo synthetically Active Radiation
CID: Carbon Isotope Discrimination
