# Supplementary material for: Transgenic chickpea (Cicer arietinum L.) harbouring AtDREB1a are physiologically better adapted to water deficit
Source: BMC Plant Biol. 2021 Jan 11;21:39. doi: 10.1186/s12870-020-02815-4 (PMC7802217; doi:10.1186/s12870-020-02815-4)
Supplement: Supplementary file 1 — Additional file 1: Sup Fig. 1. T-DNA of the AtDREB1a construct. Sup Fig. 2. Different stages of genetic transformation in chickpea (C. arietinum L.), A. De-coated DCP 92–3 seed, B. Prepared CWHEA explant, C. Explants dipped in Agrobacterium suspension, D. Explants in Whatman filter paper, E. Co-cultivated explants in SIM 1 media, F. Germinated explants in SIM 2 media, G. Explant having multiple shoots, H. Kanamycin resistant shoot (Ready for grafting), I. Micrografting, J. Mature fertile plant. Sup Fig. 3. Full image of Southern Blot analyses (1F). Sup Fig. 4. Full image of RT-PCR of transgenic chickpea lines and control (1G). (PPT 3221 kb) [file 12870_2020_2815_MOESM1_ESM.ppt]

## Slide 1
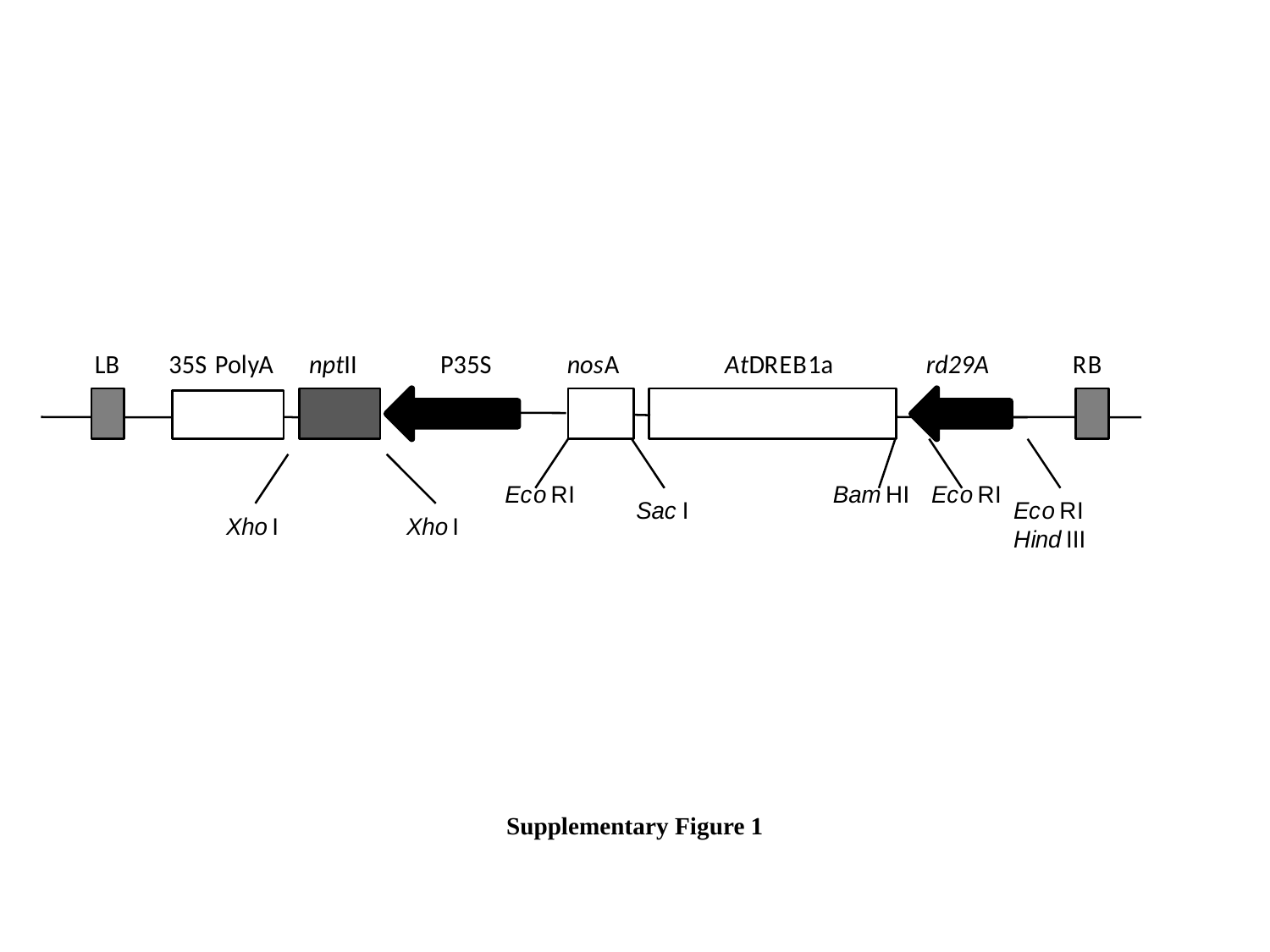

Supplementary Figure 1

## Slide 2
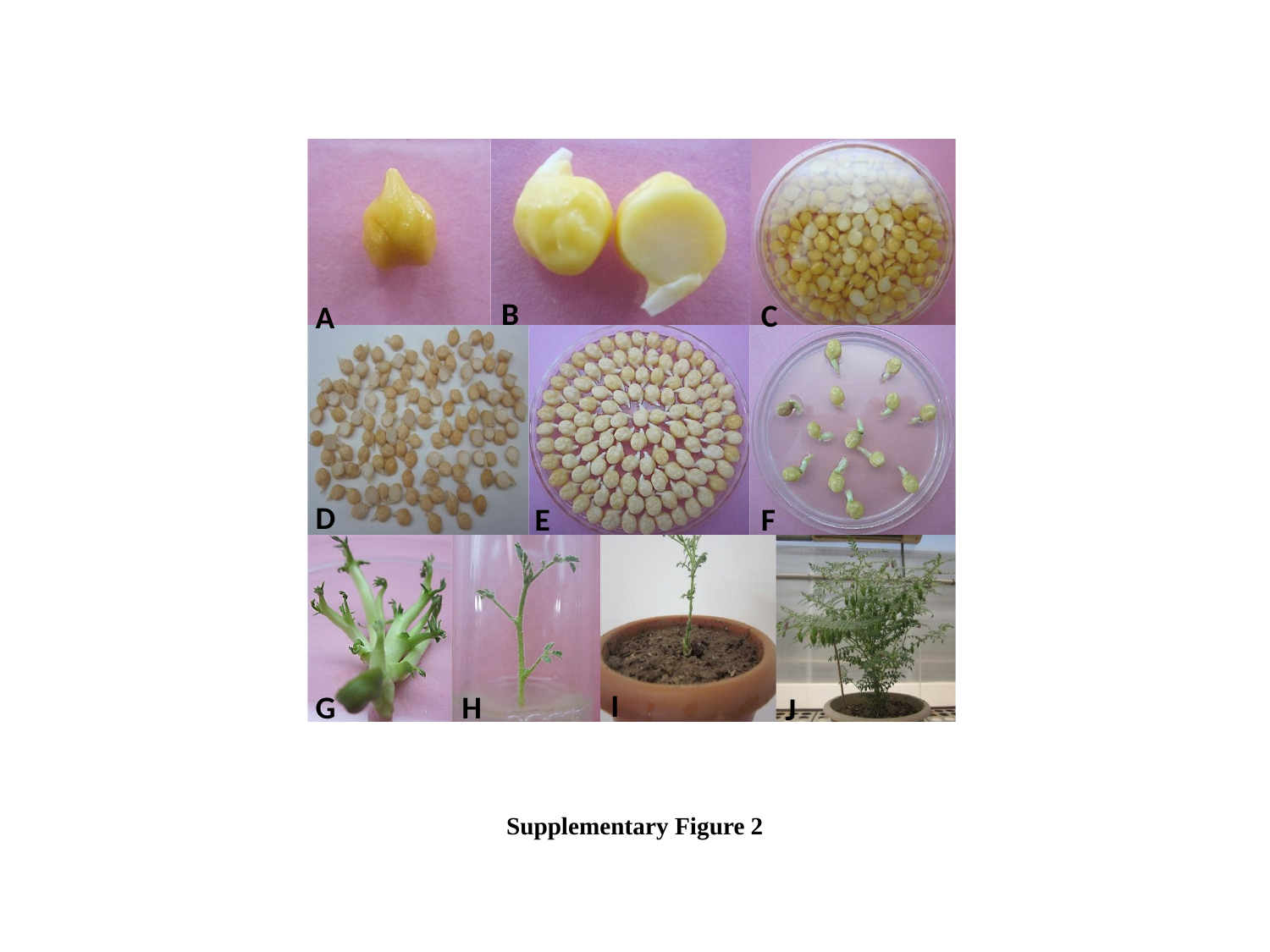

B
C
A
D
E
F
I
G
H
J
Supplementary Figure 2

## Slide 3
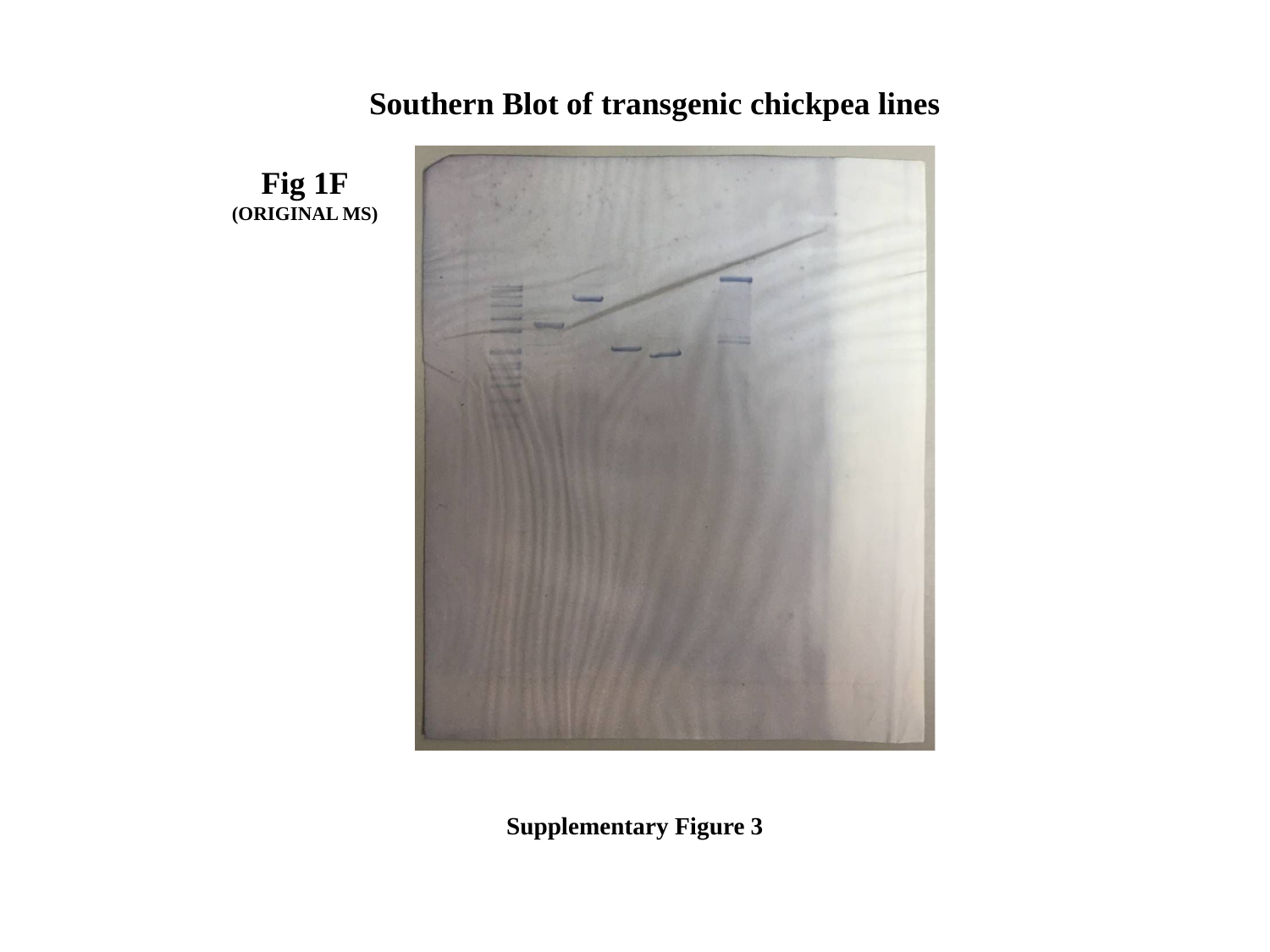

Southern Blot of transgenic chickpea lines
Fig 1F
(ORIGINAL MS)
Supplementary Figure 3

## Slide 4
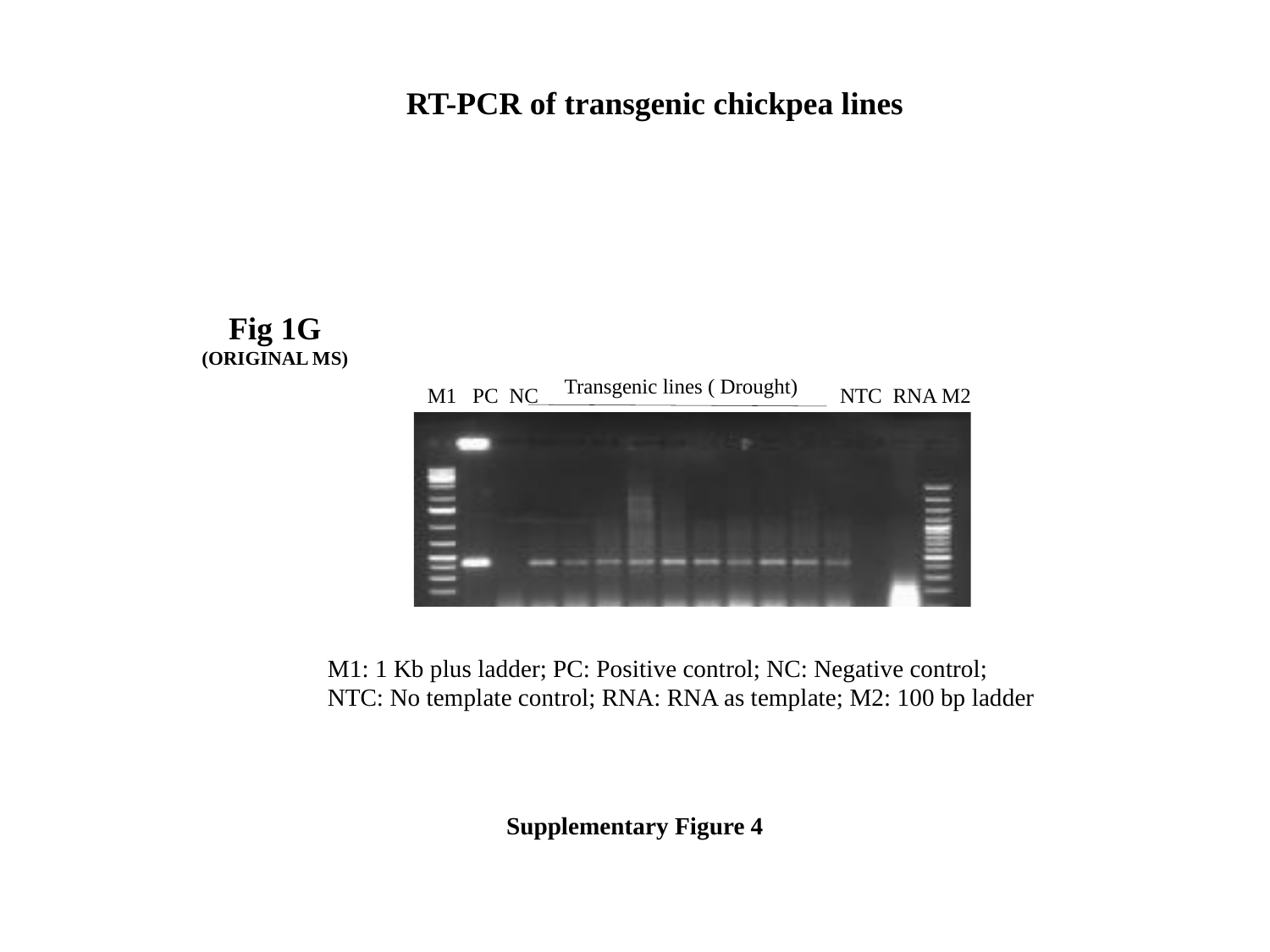

RT-PCR of transgenic chickpea lines
Fig 1G
(ORIGINAL MS)
 Transgenic lines ( Drought)
 M1 PC NC NTC RNA M2
M1: 1 Kb plus ladder; PC: Positive control; NC: Negative control; NTC: No template control; RNA: RNA as template; M2: 100 bp ladder
Supplementary Figure 4
