# Supplementary material for: Transgenic chickpea (Cicer arietinum L.) harbouring AtDREB1a are physiologically better adapted to water deficit
Source: BMC Plant Biol. 2021 Jan 11;21:39. doi: 10.1186/s12870-020-02815-4 (PMC7802217; doi:10.1186/s12870-020-02815-4)
Supplement: Supplementary file 2 — Additional file 2: Sup Table 1. Transgenic chickpea lines and Generation Advancement. Sup Table 2. Segregation of AtDREB1a in T1 progenies of four independent chickpea events. Sup Table 3. ANOVA for RWC and OA of transgenic event E5 (T2). Sup Table 4. ANOVA for RWC and OA of transgenic event E17 (T2). Sup Table 5. ANOVA for RWC and OA of transgenic event E19 (T2). Sup Table 6. ANOVA for RWC and OA of transgenic event E22 (T2). Sup Table 7. ANOVA for RWC of transgenic chickpea lines (T3). Sup Table 8. ANOVA for OP of transgenic chickpea lines (T3). Sup Table 9. ANOVA for MSI of transgenic chickpea lines (T3). Sup Table 10. ANOVA for chlorophyll content (SPAD values) of transgenic chickpea lines (T3). Sup Table 11. ANOVA for ETR of transgenic chickpea lines (T3). Sup Table 12. ANOVA for CID of transgenic chickpea lines (T3). Sup Table 13. ANOVA for seed yield of transgenic chickpea lines, under WW and WS conditions (T3). [file 12870_2020_2815_MOESM2_ESM.doc]

Supplementary Table 1: Transgenic Chickpea Lines and Generation Advancement

| **T_0_**  **lines** | **T_1_**  **seeds** | **Segregation Ratio**  **[PCR(+): PCR(-)]** | **T_2_**  **seeds** |  | **T_3_**  **seeds** |
| --- | --- | --- | --- | --- | --- |
| E_5_ | 10 | 4:6 | 60 (4 PCR positive plants) |  | NA |
| E_17_ | 12 | 9:3 | 133 (9 PCR positive plants) |  | 1371 seeds^$^ |
| E_19_ | 7 | 3:4 | 43 (3 PCR positive plants) |  | NA |
| E_22_ | 11 | 3:8 | 46 (3 PCR positive plants) |  | NA |

^$^ Phenotyping studies conducted

NA: Not Advanced

**Supplementary Table 2: Segregation of *AtDREB1a* in T_1_** progenies of four independent chickpea events

| **T_0_ lines** | **Total T_1_ seeds harvested** | **Copy number*** | **PCR (+) progenies** | **PCR (-) progenies** | **Observed ratio** | **Chi-square value^** |
| --- | --- | --- | --- | --- | --- | --- |
| E_5_ | 10 | 1 | 4 | 6 | 2:3 | 6.533 |
| E_17_ | 12 | 1 | 9 | 3 | 3:1 | 0 |
| E_19_ | 7 | 1 | 3 | 4 | 3:4 | 3.856 |
| E_22_ | 11 | 1 | 3 | 8 | 3:8 | 13.36 |

*Based on Southern Blot data

^Against 3:1 (Critical chi square at 0.01; df: 1; Value = 2.71)

**Supplementary Table 3. ANOVA for RWC and OA of transgenic event E5 (T_2_)**

**Character RWC**

| **Source of Variation** | **DF** | **Sum of Squares** | **Mean Squares** | **F-Calculated** | **Significance** |
| --- | --- | --- | --- | --- | --- |
| **Treatment** | 9 | 1,760.15 | 195.572 | 15.408 | 0.000 |
| **Error** | 40 | 507.7 | 12.692 |  |  |
| **Total** | 49 | 2,267.85 |  |  |  |

**Character OA**

| **Source of Variation** | **DF** | **Sum of Squares** | **Mean Squares** | **F-Calculated** | **Significance** |
| --- | --- | --- | --- | --- | --- |
| **Treatment** | 9 | 2.381 | 0.265 | 8.811 | 0.000 |
| **Error** | 40 | 1.201 | 0.03 |  |  |
| **Total** | 49 | 3.582 |  |  |  |
|  |  |  |  |  |  |

**Supplementary Table 4. ANOVA for RWC and OA of transgenic event E17 (T_2_)**

Character RWC

| **Source of Variation** | **DF** | **Sum of Squares** | **Mean Squares** | **F-Calculated** | **Significance** |
| --- | --- | --- | --- | --- | --- |
| **Treatment** | 10 | 3,021.94 | 302.194 | 23.89 | 0 |
| **Error** | 44 | 556.572 | 12.649 |  |  |
| **Total** | 54 | 3,578.52 |  |  |  |

Character OA

| **Source of Variation** | **DF** | **Sum of Squares** | **Mean Squares** | **F-Calculated** | **Significance** |
| --- | --- | --- | --- | --- | --- |
| **Treatment** | 10 | 7.41 | 0.741 | 13.856 | 0 |
| **Error** | 44 | 2.353 | 0.053 |  |  |
| **Total** | 54 | 9.763 |  |  |  |

**Supplementary Table 5. ANOVA for RWC and OA of transgenic event E19 (T_2_)**

Character RWC

| **Source of Variation** | **DF** | **Sum of Squares** | **Mean Squares** | **F-Calculated** | **Significance** |
| --- | --- | --- | --- | --- | --- |
| **Treatment** | 9 | 1,416.59 | 157.398 | 11.935 | 0 |
| **Error** | 40 | 527.524 | 13.188 |  |  |
| **Total** | 49 | 1,944.11 |  |  |  |
|  |  |  |  |  |  |
| **Character OA** |  |  |  |  |  |
|  |  |  |  |  |  |
| **Source of Variation** | **DF** | **Sum of Squares** | **Mean Squares** | **F-Calculated** | **Signficance** |
| **Treatment** | 9 | 3.011 | 0.335 | 10.557 | 0 |
| **Error** | 40 | 1.268 | 0.032 |  |  |
| **Total** | 49 | 4.279 |  |  |  |

**Supplementary Table 6. ANOVA for RWC and OA of transgenic event E22 (T_2_)**

**Character RWC**

| **Source of Variation** | **DF** | **Sum of Squares** | **Mean Squares** | **F-Calculated** | **Significance** |
| --- | --- | --- | --- | --- | --- |
| **Treatment** | 9 | 1,895.61 | 210.623 | 9.981 | 0 |
| **Error** | 40 | 844.096 | 21.102 |  |  |
| **Total** | 49 | 2,739.71 |  |  |  |
|  |  |  |  |  |  |
| **Character OA** |  |  |  |  |  |
| **Source of Variation** | **DF** | **Sum of Squares** | **Mean Squares** | **F-Calculated** | **Significance** |
| **Treatment** | 9 | 3.417 | 0.38 | 8.704 | 0 |
| **Error** | 40 | 1.745 | 0.044 |  |  |
| **Total** | 49 | 5.162 |  |  |  |
|  |  |  |  |  |  |

**Supplementary Table 7: ANOVA for RWC of transgenic chickpea lines**

| **Source** | **df** | **Mean Square** | **F** |  | **Significance** |  |
| --- | --- | --- | --- | --- | --- | --- |
| Transgenic lines | 15 | 324.84 | 5.53 |  | 0.0001 |  |
| Stress | 3 | 638.47 | 10.87 |  | 0.0001 |  |
| Transgenic lines * Stress | 45 | 87.36 | 1.49 |  | 0.0001 |  |
| Error | 128 | 58.71 |  |  | 0.044 |  |

Main effects (transgenic chickpea lines and stress) and interaction (transgenic lines X stress) are significant at 1% level

**Supplementary Table 8: ANOVA for OP of transgenic chickpea lines**

| **Source** | **Sum of Squares** | **df** | **Mean Square** | **F** | **Sig.** |  |
| --- | --- | --- | --- | --- | --- | --- |
| Transgenic lines | 4.511 | 15 | 0.301 | 15.113 | 0.000 |  |
| Stress | 0.472 | 1 | 0.472 | 23.708 | 0.000 |  |
| Transgenic lines * Stress | 0.931 | 15 | 0.062 | 3.119 | 0.007 |  |
| Error | 1.572 | 79 | 0.020 |  |  |  |
| LSD at 5%, 0.18 |  |  |  |  |  |  |

Main effects (transgenic chickpea lines and stress) and interaction (transgenic lines X stress) are significant at 1% level

**Supplementary Table 9: ANOVA for MSI of transgenic chickpea lines**

|  | **Source** | **Sum of Squares** | **df** | **Mean Square** | **F** | **Sig.** |  |
| --- | --- | --- | --- | --- | --- | --- | --- |
|  | Transgenic lines | 1304.93 | 15 | 87.00 | 3.03 | 0.0001 |  |
|  | Stress level (WW *vs* WS) | 6743.55 | 1 | 6743.55 | 690.87 | 0.0001 |  |
|  | Stress * transgenic lines | 2913.67 | 30 | 97.12 | 9.95 | 0.0001 |  |
|  | Error | 605.18 | 62 | 9.76 |  |  |  |

Main effects (transgenic chickpea lines and stress) and interaction (transgenic lines X stress) are significant at 1% level

**Supplementary Table 10: ANOVA for chlorophyll content (SPAD values) of transgenic chickpea lines**

| **Source** | **Sum of Squares** | **df** | **Mean Square** | **F** | **Sig.** |
| --- | --- | --- | --- | --- | --- |
| Model | 279157.94 | 64 | 4361.84 | 85.41 | 0.000 |
| Transgenic lines | 2572.02 | 15 | 171.47 | 3.36 | 0.000 |
| Stress | 41102.48 | 3 | 13700.83 | 268.27 | 0.000 |
| Transgenic lines x Stress | 7107.22 | 45 | 157.94 | 3.09 | 0.000 |
| Error | 6537.04 | 128 | 51.07 |  |  |

Main effects (transgenic chickpea lines and stress) and interaction (transgenic lines X stress) are significant at 1% level

**Supplementary Table 11: ANOVA for ETR of transgenic chickpea lines**

| **Source** | **Sum of Squares** | **df** | **Mean Square** | **F** | **Sig.** |
| --- | --- | --- | --- | --- | --- |
| Light | 6169.63 | 1 | 6169.63 | 943.65 | 0.000 |
| Transgenic lines | 3363.01 | 15 | 224.20 | 34.29 | 0.000 |
| Light x Transgenic lines | 2007.93 | 15 | 133.86 | 20.47 | 0.000 |
| Error | 418.43 | 64 | 6.54 |  |  |

Main effects (transgenic chickpea lines and stress) and interaction (transgenic lines X stress) are significant at 1% level

**Supplementary Table 12: ANOVA for CID of transgenic chickpea lines**

|  | **Source** | **Sum of Squares** | **df** | **Mean Square** | **F** | **Sig.** |  |
| --- | --- | --- | --- | --- | --- | --- | --- |
|  | Transgenic lines | 63.02 | 15 | 4.20 | 23.72 | 0.000 |  |
|  | Stress | 0.54 | 1 | 0.54 | 3.07 | 0.000 |  |
|  | Transgenic lines X Stress | 13.82 | 16 | 0.86 | 4.88 | 0.000 |  |
|  | Error | 10.98 | 62 | 0.18 |  |  |  |

Main effects (transgenic chickpea lines and stress) and interaction (transgenic lines X stress) are significant at 1% level

**Supplementary Table 13: ANOVA for seed yield of transgenic chickpea lines under WW and WS conditions**

| **Source of Variation** | **df** | **Sum of Squares** | **Mean Squares** | **F** | **Significance** |
| --- | --- | --- | --- | --- | --- |
| **Treatment (Stress level WW and WS)** | 1 | 317.596 | 317.596 | 444.606 | 0.000 |
| **Treatment (Transgenic lines and control)** | 16 | 50.61 | 3.163 | 4.428 | 0.000 |
| **Interaction**  **Stress level X Transgenic lines and control** | 16 | 100.356 | 6.272 | 8.781 | 0.000 |
| **Error** | 136 | 97.149 | 0.714 |  |  |
| **Total** | 169 | 565.711 |  |  |  |
